# Synthesis of Novel Zwitterionic Surfactants: Achieving Enhanced Water Resistance and Adhesion in Emulsion Polymer Adhesives

**DOI:** 10.3390/polym16243504

**Published:** 2024-12-17

**Authors:** Mai Toan, Jaehyouk Choi, Hang Thi Ngo, Jin-Young Bae, Seunghan Shin, Kiok Kwon

**Affiliations:** 1 Green and Sustainable Materials R&D Department, Korea Institute of Industrial Technology (KITECH), Cheonan 31056, Republic of Korea; maitoan@kitech.re.kr (M.T.); sia990709@kitech.re.kr (J.C.); ngohang@kitech.re.kr (H.T.N.); 2 School of Chemical Engineering, Sungkyunkwan University, Suwon 16419, Republic of Korea; b521@skku.edu; 3 Department of Green Process and System Engineering, University of Science & Technology (UST), Daejeon 34113, Republic of Korea

**Keywords:** zwitterionic surfactant, water born latex, emulsion adhesive, water resistance, bio-derived surfactant

## Abstract

Recent advancements in polymer materials have enabled the synthesis of bio-based monomers from renewable resources, promoting sustainable alternatives to fossil-based materials. This study presents a novel zwitterionic surfactant, SF, derived from 10-undecenoic acid obtained from castor oil through a four-step reaction, achieving a yield of 78%. SF has a critical micelle concentration (CMC) of 1235 mg/L, slightly higher than the commercial anionic surfactant Rhodacal DS-4 (sodium dodecyl benzene sulfonate), and effectively stabilizes monomer droplets, leading to excellent conversion and stable latex formation. The zwitterionic groups in SF enhance adhesion to hydrophilic substrates (glass, stainless steel, and skin). Films produced with SF exhibit outstanding water resistance, with only 18.48% water uptake after 1800 min, compared to 81% for the control using Rhodacal DS-4. Notably, SF maintains low water uptake across various concentrations, minimizing water penetration. Thus, the synthesized SF demonstrates improved adhesive properties and excellent water resistance in emulsion polymerization applications, highlighting its potential as a sustainable, high-performance alternative to petrochemical surfactants.

## 1. Introduction

In the context of climate change and the depletion of natural resources, the search for sustainable solutions has become more urgent than ever. One of these solutions is the use of bio-based materials. These materials are those produced from renewable resources, such as plants, animals, microorganisms, and organic waste. They not only help reduce dependence on fossil fuels but also contribute to environmental protection. Recent research on polymer materials has shown significant advancements in synthesizing bio-based monomers. These monomers, derived from renewable resources, are being used to create sustainable polymers that can replace traditional fossil-based materials [1]. Among the polymer synthesis methods, emulsion polymerization is considered the most environmentally friendly because it avoids the use of organic solvents. Emulsion polymerization uses water as the reaction medium, helping to minimize negative impacts on the environment and human health. This method also allows for better control over the properties of the polymer, such as particle size and dispersion, resulting in high-quality and stable products [2]. To carry out emulsion polymerization, the use of surfactants is indeed essential. Surfactants help to stabilize the emulsion by reducing the surface tension between the monomer droplets and the water phase. This stabilization is crucial for forming and maintaining the small polymer particles throughout the polymerization process. Indeed, there has been considerable research on synthesizing bio-based surfactants. Furthermore, these surfactants can potentially offer beneficial properties, such as biodegradability, biocompatibility, and mildness to the skin [3]. Recently, there have been several publications about the synthesis of surfactants from bio-derived material, such as from fatty acids [4], carbohydrates [5], amino acids [6], and soybean oil [7]. Most published bio-derived surfactants are either non-ionic or anionic. There are several recent studies on the successful synthesis of bio-based surfactants with zwitterionic structures from amino acids [8] or sulfonic zwitterionic surfactants [3]. These zwitterionic surfactants are highly valued for their safety, biodegradability, and biocompatibility [9].

The castor plant (Ricinus communis), a member of the Euphorbiaceae family, produces seeds that contain up to 50% castor oil by weight. This oil is easily extracted and finds applications in various sectors, including medicine, the chemical industry, and other technologies [10]. Several publications have discussed the use of castor oil as a starting material for the synthesis of surfactants. In 2015, Qi-Qi Zhang published a paper on the synthesis of a zwitterionic surfactant derived from castor oil and its performance evaluation for oil recovery [11]. More recently, in 2022, Abdolreza Farhadian introduced a process for the sulfonation of castor oil, utilizing this bio-surfactant to improve methane storage [12]. To the best of my knowledge, there have been very few publications about the synthesis of surfactants from castor oil for use in emulsion polymerization.

Pressure-sensitive adhesives (PSAs), also known as self-adhesives, are used in products such as tapes, label papers, and sheets. Most acrylate-based pressure-sensitive adhesives (PSAs) are synthesized from acrylate/methaacrylate through solution polymerization using ethyl acetate as a solvent [13], bulk polymerization under UV radiation [14], or via emulsion polymerization. For emulsion polymerization, most surfactants used are fossil-based, like SDS [15] or Rhodacal [16]. There have been several publications on using bio-based surfactants for emulsion polymerization. For example, Jing Hu reported the synthesis of an anionic surfactant from ω-Hydroxy Fatty Acids. However, this surfactant showed low solubility in water, limiting its application in emulsion polymerization (the solid content for polymerization is only 10%) [17]. Similarly, Bernhard V.K.J. Schmidt synthesized a non-ionic lignin-based surfactant for the emulsion polymerization of styrene, but the resulting solid content was only up to 21% [18]. Until now, no bio-derived surfactant has demonstrated fully compatible properties when compared with petroleum-based surfactants such as SDS (sodium dodecyl sulfate) or Rhodacal DS-4. Moreover, very few studies have focused on the synthesis of zwitterionic surfactants from bio-derived sources. In our study, we successfully synthesized novel zwitterionic surfactants with imidazolium and sulfonate head groups from bio-based materials, achieving a high yield. These zwitterionic compounds allow for efficient synthesis and straightforward purification. In contrast, compounds with complex synthesis and low yield pose technical challenges for replacing existing commercial surfactants. Additionally, they are suitable for both acidic and basic environments, and the excellent water solubility of sulfonate zwitterionic compounds provides a significant advantage as polar head groups in surfactants. Thus, we focused on synthesizing an SF that incorporates imidazolium and sulfonate zwitterionic functional groups. This surfactant has been used in the emulsion polymerization of butyl acrylate, hydroxyethyl acrylate, methacrylic acid, and methyl methacrylate to produce PSAs with a solid content of up to 55%. The commercial surfactant Rhodacal was used to compare the efficiency of this bio-based surfactant, and the performance of this bio-derived surfactant is comparable with Rhodacal DS-4. Furthermore, by forming interactive bonding between this zwitterionic surfactant and the polymer chains, the water resistance of the resulting PSA is significantly enhanced. As a result, the use of PSA synthesized from this surfactant shows great potential for applications in industries where PSA is exposed to high-humidity environments.

## 2. Experimental

### 2.1. Materials

10-Undecenoic acid (98%), Pd/C (10 wt.% loading), methanol (≥99.8%), thionyl chloride (≥99.0%), dimethylformamide (DMF, ≥99.8%), methylene chloride (≥99.8%), 1-(3-aminopropyl)imidazole (≥97%), ethyl acetate (EA, ≥ 99.5%), acetonitrile (≥99.9%), 1,3-propane sultone (≥99%), methacrylic acid (MAA, ≥99%), 2-hydroxyethyl acrylate, (2-HEA, ≥96%), n-dodecyl mercaptan (n-DDM, ≥98%), methyl methacrylate (MMA, ≥99%), sodium persulfate (SPS, ≥98%), tert-butyl hydroperoxide (TBHP, 70 wt% in H_2_O), ammonia solution (28.0–30.0%), KBr (≥99%), CDCl_3_ (≥99%), DMSO-d6 (99%) were purchased from Sigma–Aldrich (St. Louis, MO, USA) and directly used without purification. Butyl acrylate (BA, 99%) was purchased from DAEJUNG (Siheung-si, Gyeonggi-do, Republic Korea) and directly used without purification. Rhodacal DS-4 (sodium dodecyl benzene sulfonate, 23 wt% in H_2_O) was supplied by Rhodia Sovay Group (Banksmeadow, New South Wales, Australia).

### 2.2. Synthesis of Surfactant Novel Zwitterionic Surfactant (SF)

The surfactant SF was synthesized by the flowing procedure: 
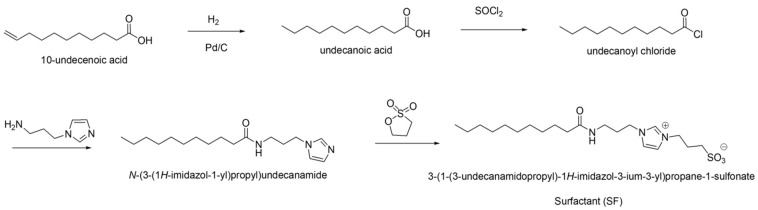


#### 2.2.1. Synthesis of Undecanoic Acid

An amount of 5 g of 10-undecenoic acid was dissolved in 50 mL of methanol. Subsequently, 0.5 g of Pd/C (10 wt% Pd on carbon, wet state 50%) was added. The reaction was carried out under a hydrogen atmosphere provided by a hydrogen balloon at room temperature for 24 h. After the reaction was complete, the mixture was filtered, and the methanol was removed using a rotary evaporator. This process yielded 4.8 g of the product in a liquid state, with a yield of 96%.

^1^H NMR (300 MHz, CDCl_3_, δ): 2.36–2.30 (2H), 1.73–1.63 (2H), 1.38–1.18 (14H), 0.89–0.82 (3H).

#### 2.2.2. Synthesis of Undecanoyl Chloride

An amount of 20 g of undecenoic acid was added to a 500 mL round-bottom flask, followed by the addition of 200 mL of thionyl chloride and three drops of DMF as a catalyst. The reaction was carried out overnight. After completion, the SOCl_2_ was removed by rotary evaporation, and any remaining SOCl_2_ was completely removed using a high vacuum. This process yielded g of a dark brown liquid with a yield of 93%.

^1^H NMR (300 MHz, CDCl_3_, δ): 2.89–2.82 (2H), 1.67–1.56 (2H), 1.38–1.16 (14H), 0.89–0.83 (3H).

#### 2.2.3. Synthesis of N-(3-(1H-imidazol-1-yl)undecanamide

A solution of 15.0 g of 10-undecenoyl chloride was dissolved in 300 mL of methylene chloride (MC). In a 1000 mL round-bottom flask (RBF) with a single neck, 36.8 g of 1-(3-aminopropyl)imidazole was added, followed by 300 mL of MC. The 10-undecenoyl chloride solution was then added dropwise to the reactor slowly. A white powder was produced during the reaction. The mixture was stirred overnight. After the reaction was complete, the mixture was filtered, and silica gel was added during the rotary evaporation process. The product was obtained via flash column chromatography (100% ethyl acetate to 100% methanol). After drying under high vacuum, 20.7 g of a white solid product was obtained, with a yield of 96%.

^1^H NMR (300 MHz, CDCl_3_, δ): 7.50–7.40 (1H), 7.10–6.90 (2H), 4.05–3.90 (2H), 3.35–3.15 (2H), 2.20–2.10 (2H), 2.05–1.90 (2H), 1.70–1.50 (2H), 1.35–1.15 (14H), 0.94–0.77 (3H).

#### 2.2.4. Synthesis of Surfactant SF

**Figure 1:**
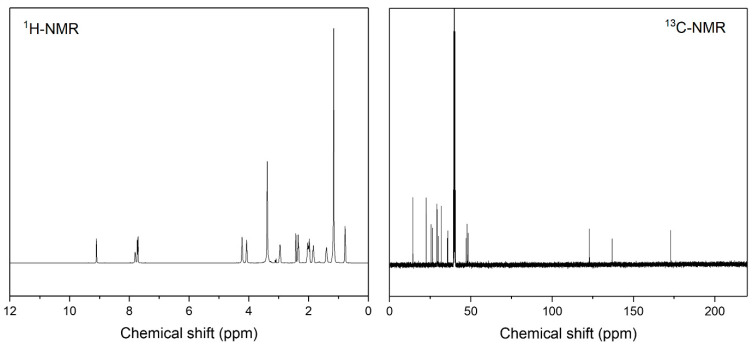
1H NMR and 13C NMR spectra of SF.

An amount of 20.7 g of N-(3-(1H-imidazol-1-yl)undecanamide was weighed, and 400 mL of acetonitrile was added, followed by 27.5 g of 1,3-propane sultone. The reaction mixture was stirred at 40 °C overnight. After the reaction was complete, silica gel was added during the removal of acetonitrile by rotary evaporation. The final product was obtained via flash column chromatography (ethyl acetate: methanol 1:1 to 100% methanol). This process yielded 26.1 g of a white solid product (surfactant, SF), with a yield of 91%. The ^1^H-NMR and ^13^C-NMR spectra of newly synthesized SF are shown in Figure 1.

^1^H-NMR (300 MHz, DMSO-d6, δ): 9.13-9.08 (s, 1H), 7.84–7.78 (s, 1H), 7.75–7.20 (s, 1H), 7.20–7.69 (s, 1H), 4.26–4.19 (t, 2H), 4.11–4.03 (t, 2H), 3.04–2.86 (m, 2H), 2.38–2.31 (t, 2H), 2.10-1.92 (m, 4H), 1.90–1.78 (m, 2H), 1.45–1.35 (m, 2H), 1.25-1.05 (m, 14H), 0.80-0.74 (t, 3H).

^13^C-NMR (300 MHz, DMSO-d6, δ): 172.80 (1C), 136.80 (1C), 122.90 (1C), 122.80 (1C), 48.30 (1C), 47.70 (1C), 47.10 (1C), 35.81 (1C), 35.55 (1C), 31.70 (1C), 30.06 (1C), 29.38 (1C), 29.35 (1C), 29.20 (1C), 29.13 (1C), 29.09 (1C), 26.52 (1C), 25.63 (1C), 22.49 (1C), 14.36 (1C).

IR (cm^−1^): NH (3450), C=O (1650), S=O (1180, 1040).

### 2.3. Synthesis of Acrylic Pressure-Sensitive Adhesion

The pre-emulsion was prepared via stirring an amount of surfactant in Table 1 and Table 2 with DW (59 g); then, 3.3 g of MAA, 5.08 g of 2-HEA, 3.56 g of MMA, and 115.06 g of BA was drop-wised added, respectively. After adding, pre-emulsion solution has been keep stirring for 30 min. Then, SPS (0.56 g) and 41.47 g of DW was introduced into the flask, which was dipped in a heated batch at 80 °C and kept at the temperature constant for 10 min. The polymerization was conducted by drop-wise addition of the pre-emulsion into the system, which lasted about 3 h. After adding the pre-emulsion solution, the reaction was maintained for an additional 3 h at 80 °C. To remove unreacted monomers, the reactants were heated to 60 °C, and 0.0356 g of THHP was added and stirred for one hour.

### 2.4. Characterization

#### 2.4.1. Spectroscopic Analysis

A spectroscopic analysis of ^1^H-NMR and ^13^C-NMR spectra was conducted using the Bruker Avance 300 NMR device (Karlsruhe, Germany) with CDCl_3_ or DMSO-d6 as the solvent. Infrared spectroscopy was performed with a Nicolet 6700 instrument from Thermo Scientific (Waltham, MA, USA), where the cured samples were pulverized, combined with KBr, and compressed into pellets.

#### 2.4.2. Surface Tension

Surface tension measurements were conducted using a Sigma 702 Tension Meter (Biolin Scientific, Gothenburg, Västra Götaland County, Sweden) at room temperature.

#### 2.4.3. Monomer Conversion

Polymerization conversion was measured by weighing the emulsion solution, then placing it in an oven at 100 °C for 12 h. After drying, the amount of polymer was weighed, and the polymer conversion was calculated as the percentage of dried polymer in the emulsion solution.

#### 2.4.4. Particle Size

The average particle diameter was measured by dynamic light scattering (DLS) using an ELSZ-2000ZS from Otsuka Electronics (Tokushima, Tokushima Prefecture, Japan).

#### 2.4.5. Adhesion Tests

The basic adhesive properties of latex films were analyzed using a 90° peel test on glass, stainless steel, and pig skin substrates. Latex-coated PET films were cut into 25 mm wide strips and attached to glass, stainless steel, and pig skin substrates (cleaned with acetone prior to use). After 5 min, the 90° peel strength was measured using the Surta TA peel tester (CHEMILAB, Gimpo-si, Gyeonggi-do, Republic Korea) at a rate of 300 mm/min. Water absorption was tested on film specimens with a thickness of 1 mm and a diameter of 25 mm.

#### 2.4.6. Water Absorption Test

The specimens were immersed in water for a specific period at room temperature. After immersion, the specimens were removed from the water, and excess water was blotted off the surface using absorbent paper. The specimens were then weighed; water absorption was calculated as the percentage of the absorbed water mass relative to the dried sample.

#### 2.4.7. Water Contact Angle Measurement

The static contact angle (CA) of water on the film surface was measured. We cast a 30 μm film on a glass plate and allowed the film to dry overnight at room temperature. We deposited a 20 μL droplet of distilled water on the film surface and measured the static CA. For each sample, the average CA was reported from 10 measurements.

## 3. Results and Discussion

### 3.1. Surface Tension Measurement

**Figure 2:**
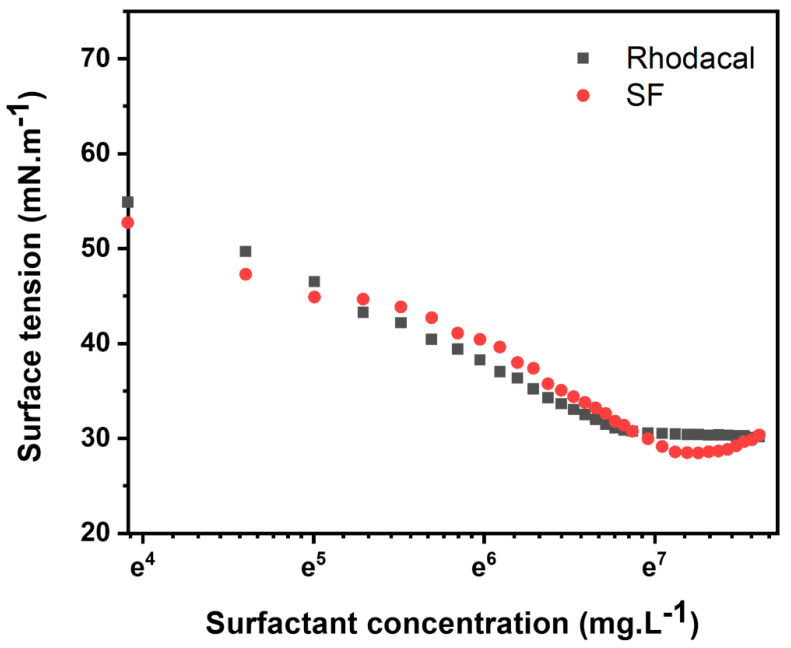
Surface tension measurement of SF and Rhodacal DS-4.

Figure 2 depicts the surface tension of two surfactants, Rhodacal DS-4 and SF, as a function of concentration at 25 °C, showing a notable decrease as surfactant concentrations increase. The critical micelle concentration (CMC) and minimun surface tension at CMC values were estimated from the breakpoints of these plots. The CMC and minimum surface tension of Rhodacal DS were 961.5 mg/L and 30.78 mN/m, respectively, while SF exhibited values of 1235 mg/L and 28.65 mN/m, respectively. The minimum surface tension values indicate the ability of the surfactants to lower surface tensions. The newly synthesized zwitterionic surfactants, SF, demonstrate a greater efficacy in lowering the surface tension of water compared to Rhodacal DS-4, attributed to its dual positive and negative charges in the head group [19,20]. This structural feature enhances interactions with polar water molecules, allowing for a more effective reduction in surface tension and exhibiting superior performance compared to anionic surfactants, which possess only a single negative charge. It is known that the presence of hydrophobic impurities could reduce the apparent break in the surface tension [21]. The SF was synthesized without impurities and measured under the same conditions as Rhodacal, indicating that the low surface tension value is attributed to structural characteristics.

The surface excess concentration at 25 °C was determined using the Gibbs adsorption isotherm equation [22]. The surface excess concentration for SF was found to be (4.30 × 10^−6^ mol/m^2^), slightly higher than the (3.43 × 10^−6^ mol/m^2^) for Rhodacal DS-4, indicating that SF requires more surfactant to saturate a given surface area [23]. Additionally, the minimum area occupied by each monomer for SF is (38.7 Å^2^), which is lower than the (48.5 Å^2^) for Rhodacal DS-4.

### 3.2. Preparation and Characterization of Emulsion Polymerization Using New SF Surfactant

**Figure 3:**
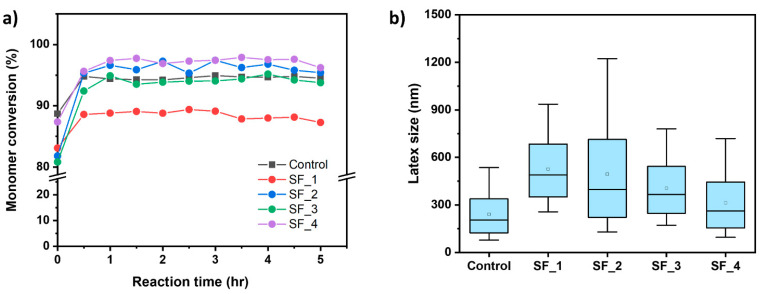
(**a**) Final monomer conversion and (**b**) particle size of emulsion polymerization.

Figure 3 presents the monomer conversion as a function of polymerization time and the particle size of the resulting latex in emulsion polymerization processes with varying concentrations of SF. This polymerization was carried out using a semi-continuous method, with a feeding time of 3 h. At the start of the reaction (0 h), immediately after the feeding began, over 80% of the monomer was consumed during the feeding period. After 2 h of the reaction, the monomer conversion reached a saturated state. As shown in Figure 3a, for both the control sample and the system using SF at concentrations above 2.18%, the final conversion exceeded 95%. In the case where SF was used at 1.45%, the final conversion was less than 90%, which may be due to an insufficient surfactant amount, resulting in poor dispersion of the monomer into the droplets and leading to lower overall conversion. The newly synthesized SF demonstrates monomer conversion that is comparable to or exceeds that of the commercial surfactant Rhodacal when utilized at identical concentrations. Figure 3b shows that increasing the SF surfactant concentration from 0.75 wt% to 2.9 wt% reduces the latex particle size from 458.50 nm to 364.2 nm. This can be explained by the fact that, at low surfactant concentrations, the formation of micelles is limited, providing fewer nucleation sites for initiating the polymerization process, which results in larger particle sizes. Conversely, as the surfactant concentration increases, the formation of additional micelles occurs, establishing more polymerization sites and consequently leading to smaller particle sizes [24]. The type of surfactant also influences particle size. For instance, at a surfactant concentration of 2.9 wt%, the latex produced with Rhodacal DS-4 measures 223.4 nm, whereas the latex produced with SF measures 364.2 nm. The particle size distribution for each sample can be found in Figure S1. This phenomenon suggests that Rhodacal DS-4’s lower CMC leads to more nucleation sites and subsequently more latex particles during polymerization, resulting in smaller particle sizes than SF [24,25,26]. In summary, the newly synthesized SF can effectively be used in conventional emulsion polymerization, forming latex that is comparable to or better than that produced with the petrochemical-based surfactant Rhodacal DS-4.

### 3.3. Adheesive Properties

**Figure 4:**
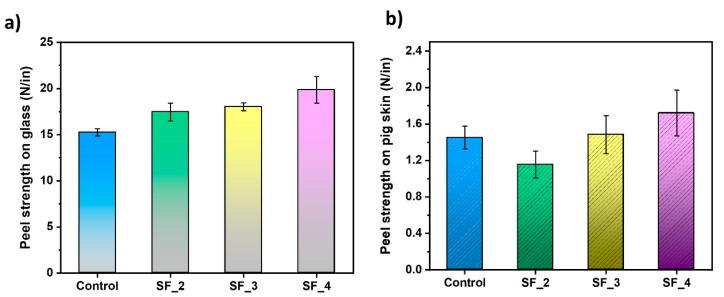
Ninety-degree peel strength of SF sample on (**a**) glass and (**b**) pig skin substrates.

An evaluation was conducted to assess the adhesion of emulsions polymerized with varying compositions of SF on different substrates (Figure 4). All emulsion solutions were coated onto 75 µm thick polyethylene terephthalate (PET) film with a uniform dry thickness of 30 μm. However, the SF_1 sample was excluded from the adhesion evaluation due to its low monomer conversion. The adhesive properties were evaluated using a 90-degree peel strength test on substrates such as glass, pig skin, and stainless steel. Notably, a detailed examination of the adhesion results on glass showed that, as the SF content increased from 1.45 wt% to 2.9 wt%, the adhesion strength of the formed SF emulsion gradually increased from 19.87 N/in to 19.87 N/in (Figure 4a). This improvement is likely due to the zwitterionic structure of the SF molecule, which enhances the interaction between the hydrophilic glass substrates and the latex as the SF concentration rises [27,28]. This trend is also observed with another hydrophilic substrate, stainless steel (Figure S2). Interestingly, as shown in Figure 4b, the zwitterionic functionality of SF latex significantly enhances adhesion to the skin. It is well known that zwitterionic functional groups provide high adhesion through interactions with skin [29,30]. The imidazole N+ group and sulfonate -SO_3_^−^ group of zwitterionic SF, which possess a strong dipole moment, can engage with polar functional groups on the skin, including -COOH, -NH_2_, -CONH, and -SH [31,32]. In contrast, only the anionic -SO_3_^−^ group of the anionic surfactant Rhodacal DS-4 contributes to interfacial adhesion. This concept is also evident in the SF latex we produced. When comparing the adhesion values of the control and SF-4 on skin, using the same surfactant content, SF-4 shows a higher value of skin adhesion. Additionally, when comparing the adhesion of latex with varying SF content, the adhesion to pig skin for SF-2, SF-3, and SF-4 was observed to consistently increase from 1.16 N/in to 1.48 N/in and 1.72 N/in, respectively. From these results, we can conclude that using zwitterionic surfactants enhances the bonding between substrates and latex, thereby increasing adhesion. Based on these results, we confirmed that the zwitterionic surfactant can enhance adhesion not only to hydrophilic surfaces like glass and stainless steel, due to various interactions at the latex interface, but also improve adhesion to the skin.

### 3.4. Water Resistance

**Figure 5:**
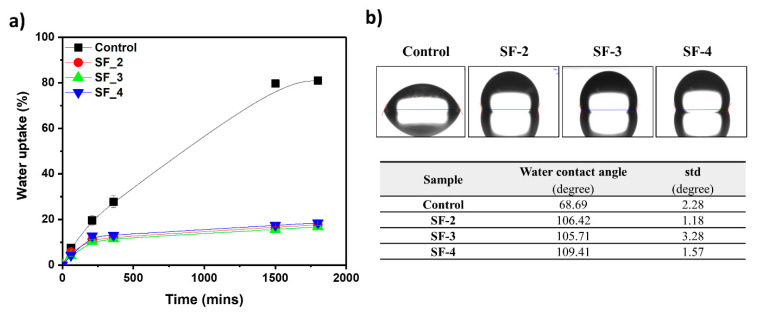
(**a**) Water uptake and (**b**) water contact angles of SF latex films.

**Figure 6:**
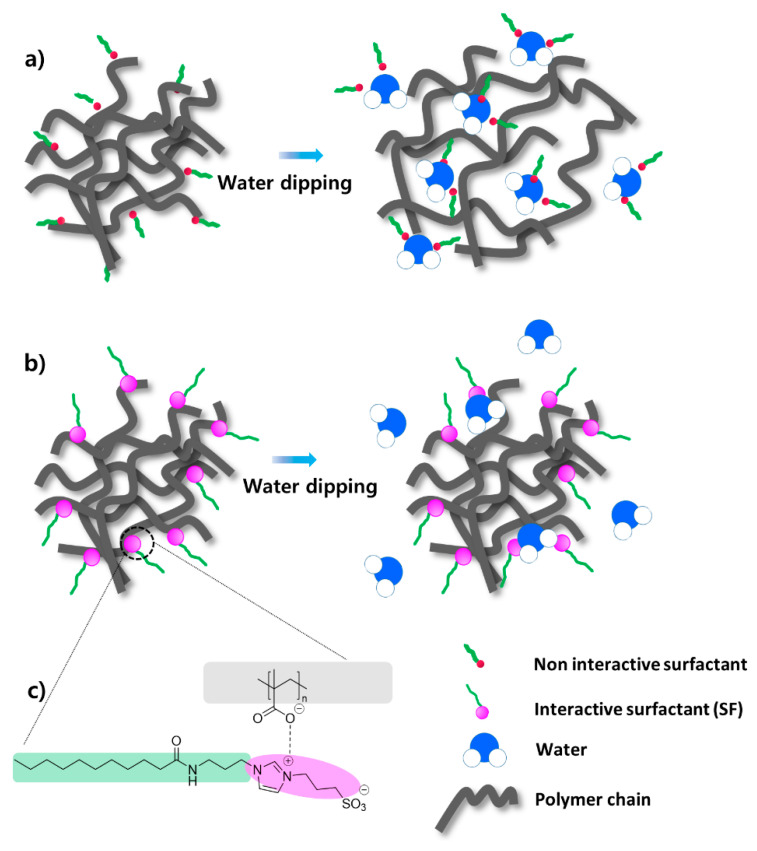
Illustrating the decease in water resistance due to the mobilization of Rhodacal DS-4 in the water phase (**a**), the penetration of SF in the polymer network (**b**), and the bonding between SF and latex (**c**).

The issue of inadequate water resistance in emulsion adhesives is largely attributed to the residual surfactant at the interfaces of emulsion particles during film formation. Resolving this issue is key to enhancing water resistance and enabling the use of emulsion adhesives with improved durability. Figure 5 shows the water absorption over immersion time for the control film with the anionic surfactant Rhodacal DS-4 and films with varying concentrations of zwitterionic SF. At 1800 min of immersion, the water uptake values for the SF-2, SF-3, and SF-4 samples were 17.62%, 16.74%, and 18.48%, respectively, significantly lower than the 81% observed for the control sample with Rhodacal DS-4. In general, for emulsion adhesives, as the surfactant content increases, the water uptake values significantly increase due to the reduction in the surface tension of water, making the absorption of water into the polymer network easier [33]. However, quite remarkably, as shown in Figure 5, in the case of the SF samples, the water uptake values remained nearly constant without any increase, even as the surfactant content increased from 1.45 wt% to 2.9 wt%, indicating that SF does not affect the water absorption of the polymer network. The contact angle values also confirmed the high water resistance when SF was used as a surfactant compared to Rhodacal DS-4. The contact angle of the control sample was 68.69°, and this value significantly increased to between 105.71° and 109.41° when SF was used as a surfactant. Furthermore, the contact angle value remained stable, even with increasing concentrations of the SF surfactant. As illustrated in Figure 6, this phenomenon can be explained by the formation of bonds between zwitterionic ions and –COO^−^ [34] after the water removal process. Due to the formation of this bond, the SF was integrated within the polymer structure, thereby losing its surfactant activity. As a result, the diffusion of water into the polymer network becomes more difficult, leading to a decrease in water uptake. As expected, when comparing the effectiveness of surfactant SF and Rhodacal DS-4, SF demonstrated superior performance in reducing water uptake compared to Rhodacal DS-4.

## 4. Conclusions

In this study, we successfully synthesized a new bio-derived surfactant, SF, from 10-undecenoic acid, featuring zwitterionic groups that enhance adhesion and water resistance in emulsion polymerization. SF outperformed the petrochemical-based surfactant Rhodacal DS-4, demonstrating better monomer conversion despite a slightly higher critical micelle concentration (CMC). Moreover, at the same concentration (2.9 wt%), SF achieved a peel strength of 19.87 N/in on glass, compared to 15.26 N/in for Rhodacal DS-4; similar improvements were observed on stainless steel and pig skin. The zwitterionic structure fosters interactive bonds between surfactant and polymer chains, promoting surfactant penetration into the polymer matrix and significantly reducing water absorption from over 80% with Rhodacal DS-4 to 18.48% with SF after 1800 min in water. This behavior results from the strong binding of zwitterionic groups to polymer chains, effectively blocking water penetration. Overall, SF demonstrates great potential as a sustainable alternative to petroleum-based surfactants in emulsion polymerization, significantly enhancing adhesion properties and water resistance while supporting the development of high-performance bio-based materials.

## Figures and Tables

**Table 1:** Sample code of latex investigated in this study and their monomer conversion and particle sizes.

Sample Code	Surfactant	Surfactant Amount	Monomer Conversion	Particle Size	Monomer
	wt%/M	g	%	nm	wt/wt
Control	2.9	3.68	93.7	223.4	BA/MAA/MMA/2-HEA = 90/3/3/4
SF_1	0.75	0.927	87.3	458.5	
SF_2	1.45	1.85	95.4	433.3	
SF_3	2.18	2.78	93.8	386.6	
SF_4	2.9	3.68	96.2	364.2	

**Table 2:** Emulsion polymerization recipe chart investigated in this study.

Reaction Step	Reagent	Amount (g)
Pre-emulsion	Surfactant	See the Table 1
	DI water	55
	MAA	3.3
	2-HEA	5.08
	MMA	3.56
	BA	115.06
Reactor	DI water	41.47
	SPS	0.56
Chaser	TBHP	0.26

## Data Availability

The original contributions presented in this study are included in the article/Supplementary Material. Further inquiries can be directed to the corresponding authors.

## Supplementary Materials

The following supporting information can be downloaded at: https://www.mdpi.com/article/10.3390/polym16243504/s1, Figure S1: Particle size distribution of Control, SF samples; Figure S2: 90° peel strength on stainless steel.
