# Supplementary material for: Synthesis of Novel Zwitterionic Surfactants: Achieving Enhanced Water Resistance and Adhesion in Emulsion Polymer Adhesives
Source: Polymers (Basel). 2024 Dec 17;16(24):3504. doi: 10.3390/polym16243504 (PMC11679416; doi:10.3390/polym16243504)
Supplement: Supplementary file 1 [file polymers-16-03504-s001.zip › polymers-3311618-supplementary.pdf]

Supplementary material

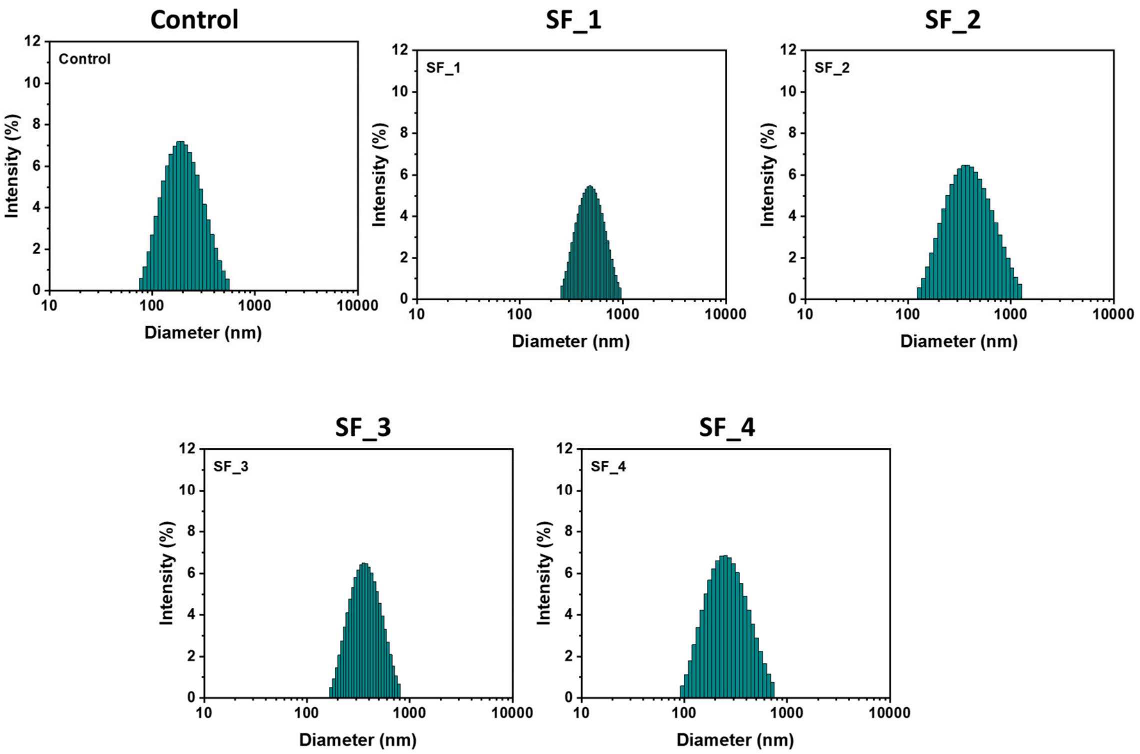

Fig. S1. Particle size distribution of Control, SF samples

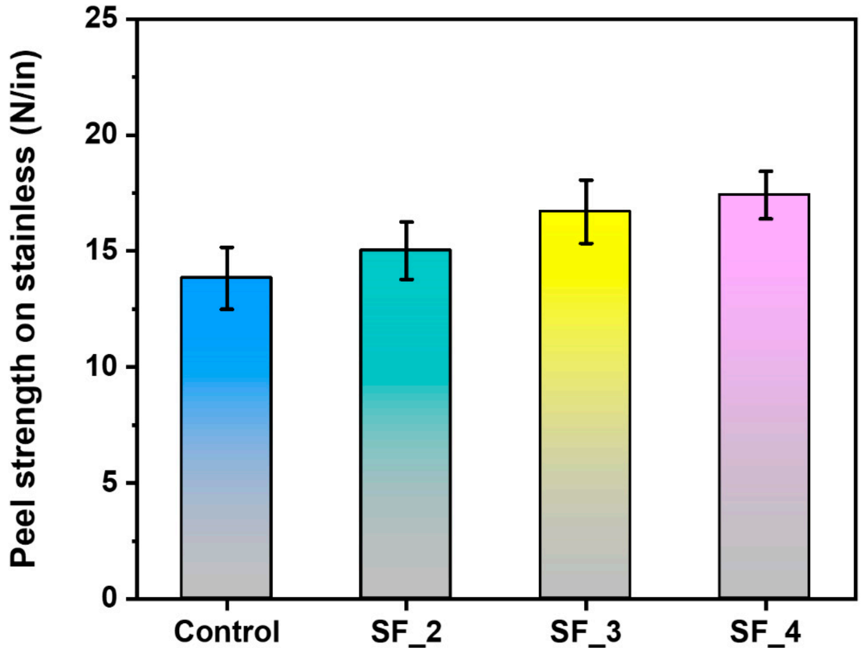

Fig. S2. 90° peel strength on stainless steel.
